# Pediatric tonsillar synovial sarcoma: extremely rare case report

**DOI:** 10.3389/fped.2025.1636916

**Published:** 2025-10-13

**Authors:** Şule Çalışkan Kamış, Begül Yağcı

**Affiliations:** Adana Faculty of Medicine, Adana City Education and Research Hospital, Department of Pediatric Hematology and Oncology, University of Health Sciences, Adana, Türkiye

**Keywords:** synovial sarcoma, tonsil, cancer, case report, pediatric cancer

## Abstract

**Background:**

Synovial sarcoma (SS) is a malignant tumor characterized by partial epithelial differentiation. While SS predominantly affects older children and young adults, it can arise in nearly any anatomical region. Its occurrence in the tonsil, particularly in the pediatric population, is extremely rare, with only one other case reported in the literature. This rarity poses diagnostic challenges, as its symptoms often resemble more common conditions such as a peritonsillar abscess. Reporting such cases is crucial to expanding the understanding of SS, refining diagnostic approaches, and guiding treatment strategies. This case offers valuable insights into the management of SS in atypical locations.

**Case presentation:**

We report the second documented case of pediatric tonsillar synovial sarcoma (SS). The patient, a 5-year-old girl, presented with a 1.5-month history of persistent fever and dysphagia. Radiological examinations revealed a mass originating from the right palatine tonsil, causing irregular narrowing of the oropharyngeal lumen, initially suspected to be a peritonsillar abscess. An incisional biopsy of the right tonsil was performed, and histopathological analysis confirmed the diagnosis of biphasic SS. The patient underwent six cycles of chemotherapy, resulting in significant tumor regression by the 6th month of treatment. This case underscores the necessity of a multidisciplinary approach, combining surgery and chemotherapy, in managing rare tumors.

**Conclusions:**

This case highlights the importance of considering rare malignancies like synovial sarcoma in the differential diagnosis of atypical tonsillar masses, especially in pediatric patients with persistent symptoms. The successful outcome achieved through a multidisciplinary approach emphasizes the need for coordinated care in managing such complex tumors. This case contributes valuable insights to the limited literature on pediatric tonsillar SS and may guide future diagnostic and therapeutic strategies for similar cases.

## Introduction

Synovial sarcoma (SS) is a malignant soft tissue tumor characterized by partial epithelial differentiation. It is most commonly seen in young individuals and can arise in almost any anatomical region (1, 2). SS accounts for approximately 10% of all soft tissue sarcomas (STS) (3, 4). In the pediatric population, SS is the most prevalent non-rhabdomyosarcoma STS (5, 6). It is often located near large joints such as the knee and ankle (7), but it can also occur in internal organs (e.g., heart, lungs, pleura, kidney), the oral cavity, mediastinum, retroperitoneum, peritoneum, central nervous system, and peripheral nerves (8). Compared to SS in other anatomical regions, head and neck synovial sarcoma (HNSS) is thought to have a higher likelihood of metastasis, typically spreading hematogenously (9, 10). Only 3%–5% of SS cases occur in the head and neck region (HNR), with SS originating from the tonsils being particularly rare; only two well-documented cases have been reported in the HNR in the English literature (11, 12). The first pediatric case of tonsillar synovial sarcoma was described by Yalçin et al. in 2020 (13). Given its rare localization, the management of tonsillar SS is primarily based on case reports (12, 13). We report what is, to the best of our knowledge, the second pediatric tonsillar SS case in the literature (14). This case, with its unusual location and treatment approach, may add valuable insight to the existing literature.

### Literature review methods

In addition to clinical management and treatment protocols, a systematic literature review was conducted to evaluate previous reports and case presentations on pediatric tonsillar synovial sarcoma (SS). The literature review was performed using multiple databases, including PubMed, Google Scholar, and Scopus. Search terms such as “pediatric tonsillar synovial sarcoma,” “tonsillar SS,” and “head and neck synovial sarcoma” were used. Inclusion criteria were based on selecting case reports, reviews, and articles published within the last 10 years that discussed treatment protocols, outcomes, and prognostic factors for pediatric tonsillar SS. Articles published in languages other than English and those not directly related to tonsillar synovial sarcoma were excluded.

The identified articles were carefully reviewed, and data regarding clinical findings, treatment strategies, histopathological features, and prognostic outcomes were extracted and analyzed. This review contributed to a deeper understanding of the rarity and treatment challenges of pediatric tonsillar synovial sarcoma by correlating our case with the current literature.

## Case report

**Figure 1:**
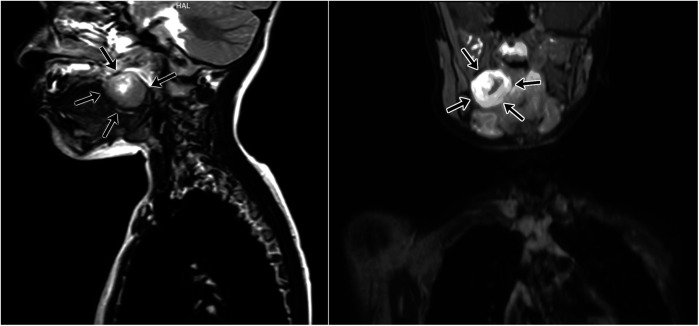
Magnetic resonance imaging (MRI) of synovial sarcoma originating from the right palatine tonsil. The tumor irregularly narrows the oropharyngeal lumen and exhibits infiltrative characteristics toward surrounding tissues. Contrast-enhanced T1-weighted images demonstrate significant contrast uptake by the lesion.

A 5-year-old girl presented with a 1.5-month history of fever and difficulty swallowing. The physical examination revealed a mass in the right tonsil that narrowed the oropharynx. The right tonsil appeared enlarged with a smooth surface and showed no signs of ulceration. Her medical history was otherwise unremarkable. Contrast-enhanced magnetic resonance imaging (MRI) revealed a solitary mass measuring 30 × 30 × 30 mm in the right lateral oropharynx, extending to the hypopharynx, and irregularly narrowing the oropharyngeal lumen. Given the absence of obvious liquid components within the mass, peritonsillitis or an early peritonsillar (non-organised) abscess was initially suspected (Figure 1). An 18F-Fluorodeoxyglucose-Positron Emission Tomography/Computed Tomography (18F-FDG-PET-CT) scan showed metabolic activity (SUV max: 5.5) in the region of the right tonsil. No distant metastases were observed on the PET scan. PET-CT was performed to exclude the possibility of systemic disease and to guide further diagnostic and therapeutic planning, as the mass was initially suspected to represent an infectious or neoplastic lesion. An incisional biopsy of the right tonsil was performed, and histopathological examination confirmed the diagnosis of biphasic synovial sarcoma.

### Pathological findings

Immunohistochemical analysis showed positive staining for Cytokeratin 7 (CK7) and EMA in the epithelial component. CD99 and bcl2 staining revealed positive results in the mesenchymal component. Both components tested positive for TLE-1 and INI-1, and negative for S100, CD34, and desmin. The Ki67 proliferation index was 25%–30%. To rule out other differential diagnoses, such as rhabdomyosarcoma and fibrosarcoma, additional immunohistochemical markers were tested. These included MyoD1 and desmin, which were negative, further supporting the diagnosis of synovial sarcoma. According to the French Federation of Cancer Centers Sarcoma Group (FNCLCC) grading system, the tumor was classified as grade 3 (differentiation: score 3, mitosis: score 3, necrosis: score 0). Due to the fragmented nature of the tissue samples, surgical margins could not be assessed.

### Treatment

Chemotherapy with ifosfamide (1.8 mg/m^2^; 3 days, every 28 days) and doxorubicin (15 mg/m^2^; 3 days, every 28 days) was initiated. The therapeutic approach was based on standard protocols for synovial sarcoma, including chemotherapy, as there were no surgical interventions performed due to the location of the tumor. We followed the treatment guidelines provided by the European Society for Medical Oncology (ESMO) for soft tissue sarcomas. The patient completed 6 cycles of chemotherapy as per the treatment protocol. After 6 cycles, an 18F-FDG-PET-CT scan showed a decrease in metabolic activity (SUV max: 5.2) in the right tonsil (suggesting a reactive change?). By the 6th month of treatment, the tumor had significantly regressed.

### Follow-up

The patient, initially diagnosed at 5 years of age, was followed regularly for 3 years. During follow-up, significant regression of the right tonsil was observed. By the 6th month of treatment, the affected tonsil had reduced in size to closely resemble the normal tonsil. Now, at 8 years and 3 months, the patient continues regular follow-up to monitor for any potential recurrence or late effects of chemotherapy.

## Discussion

Synovial sarcoma is a rare soft tissue malignancy, accounting for approximately 8%–10% of all soft tissue neoplasms (15). It typically occurs near large joints, but its presence in the head and neck—an area with limited synovial tissue—is rare (16). Given its rarity, especially in the tonsils, management of tonsillar SS primarily relies on case reports (12, 13). The first pediatric case of tonsillar SS was reported by Yalçin et al. in 2020, who described the successful surgical resection and adjuvant chemotherapy of a similar case (13). We present the second documented case of pediatric tonsillar SS, to the best of our knowledge.

Treatment for SS generally involves surgical excision with negative margins, complemented by chemotherapy and radiotherapy based on the patient's and tumor's characteristics (17). Prognosis for localized SS is generally favorable, with most patients being long-term survivors. However, those with metastatic disease have a poorer prognosis. Tumors larger than 5 cm, invasion, and a high histological grade are associated with worse outcomes (18, 19). In our case, the localized SS was smaller than 5 cm, leading us to believe the prognosis is favorable, though we continued close monitoring for potential metastasis. The role of chemotherapy in pediatric synovial sarcoma remains unclear, with some cases showing good responses only to chemotherapy without the need for surgery (20).

In typical synovial sarcoma cases, complete surgical resection with negative margins remains the cornerstone of therapy. However, in head and neck localizations, achieving clear surgical margins is often limited by anatomical constraints, which necessitates the use of multimodal approaches, including chemotherapy and radiotherapy (21).

This case contributes to the limited literature on the successful management of pediatric tonsillar synovial sarcoma with chemotherapy alone. In our patient, tonsillectomy was not performed at diagnosis due to the risk of perioperative complications, given the tumor size and infiltration of adjacent structures. Instead, a limited incisional biopsy was chosen to obtain tissue for diagnosis. Following chemotherapy, tonsillectomy was not performed as the tumor showed marked regression and complete excision would have entailed significant morbidity.

Compared with the previously reported pediatric tonsillar SS case, where surgery combined with adjuvant chemotherapy achieved remission, our case demonstrates that chemotherapy alone may also provide favorable outcomes when surgery is not feasible. This highlights the importance of tailoring treatment to tumor localization and individual patient risks.

Furthermore, although the tumor was <5 cm and localized, it was classified as FNCLCC grade 3. High-grade histology is usually associated with poorer outcomes, underscoring the need for close surveillance despite the patient's favorable clinical course.

Long-term follow-up is also essential in pediatric patients, not only for recurrence surveillance but also for monitoring potential late effects of chemotherapy, such as cardiotoxicity and impacts on growth or fertility. Multidisciplinary follow-up involving pediatric oncology, cardiology, and endocrinology teams is therefore recommended.

### Prognosis and risk stratification

In terms of prognosis, the recurrence rate for synovial sarcoma is around 15%–30%, with a higher likelihood of recurrence in patients with larger tumors, high-grade histology, and distant metastasis (22). For pediatric patients, the prognosis is generally better when the tumor is localized, small, and amenable to surgical resection. However, when surgical resection is not possible or the tumor is inoperable, as in our case, chemotherapy becomes a critical treatment modality. While there is no consensus on the exact chemotherapy regimen for pediatric SS, studies have shown that multi-agent chemotherapy, including ifosfamide and doxorubicin, is commonly employed and may yield favorable outcomes even without surgery (23).

Tumor size <5 cm and the absence of distant metastasis are considered favorable prognostic indicators. These features supported a lower risk profile in our patient. Recent reports also emphasize that, while traditional prognostic markers remain critical, emerging systemic options—including immunotherapy and targeted cellular therapies—may further influence outcomes in high-risk or advanced synovial sarcoma cases (24).

In our patient, who did not undergo surgery due to the tumor's location, chemotherapy with ifosfamide and doxorubicin was used. The absence of distant metastasis and the smaller size of the tumor were positive factors in the patient's risk stratification, indicating a lower risk of recurrence. The patient's favorable response to chemotherapy further supports the hypothesis that chemotherapy alone can be an effective treatment strategy for pediatric tonsillar synovial sarcoma, at least in localized cases.

For diagnostic work-up of head and neck synovial sarcoma, MRI is particularly useful for local extension, CT for bone involvement, and PET-CT for metabolic characterization and detection of metastasis. In our case, MRI and PET-CT were complementary in excluding abscess formation and systemic spread.

In head and neck synovial sarcoma, multimodal imaging plays a pivotal role in diagnosis and staging. MRI provides excellent soft tissue resolution, CT can detect subtle bone involvement, and PET-CT offers functional and metabolic assessment, assisting in differentiation from infectious lesions. Integrating these modalities with histopathology and molecular studies is recommended to minimize diagnostic pitfalls (25).

The current case emphasizes the importance of a tailored approach to treatment and the potential for chemotherapy alone in certain cases of pediatric SS. As more cases are documented, further studies will be needed to define clear prognostic factors and optimal treatment regimens for this rare malignancy.

## Conclusion

In conclusion, tonsillar synovial sarcoma is an extremely rare malignancy in the pediatric population. Further studies and accumulated case reports are necessary to establish evidence-based treatment strategies and prognostic factors for this tumor. We believe that the management of such rare tumors requires a multidisciplinary approach. Our case highlights the potential role of chemotherapy alone when surgery is not feasible and adds valuable insight into the limited literature on pediatric tonsillar synovial sarcoma.

## Data Availability

The raw data supporting the conclusions of this article will be made available by the authors, without undue reservation.
